# A Single Shot of Vesicles

**DOI:** 10.1264/jsme2.ME22083

**Published:** 2022-12-09

**Authors:** Masanori Toyofuku, Yousuke Kikuchi, Azuma Taoka

**Affiliations:** 1 Faculty of Life and Environmental Sciences, University of Tsukuba, Tsukuba, Ibaraki 305–0006, Japan; 2 Microbiology Research Center for Sustainability (MiCS), University of Tsukuba, Tsukuba, Ibaraki 305–0006, Japan; 3 WPI Nano Life Science Institute (WPI-NanoLSI), Kanazawa University, Kakuma-machi, Kanazawa, 920–1192, Japan; 4 Institute of Science and Engineering, Kanazawa University, Kakuma-machi, Kanazawa 920–1192, Japan

**Keywords:** membrane vesicle, bacterial communication, atomic force microscopy

## Abstract

Bacteria communicate through signaling molecules that coordinate group behavior. Hydrophobic signals that do not diffuse in aqueous environments are used as signaling molecules by several bacteria. However, limited information is currently available on the mechanisms by which these molecules are transported between cells. Membrane vesicles (MVs) with diverse functions play important roles in the release and delivery of hydrophobic signaling molecules, leading to differences in the dynamics of signal transportation from those of free diffusion. Studies on *Paracoccus denitrificans*, which produces a hydrophobic long-chain *N*-acyl homoserine lactone (AHL), showed that signals were loaded into MVs at a concentration with the potential to trigger the quorum sensing (QS) response with a “single shot” to the cell. Furthermore, stimulating the formation of MVs increased the release of signals from the cell; therefore, a basic understanding of MV formation is important. Novel findings revealed the formation of MVs through different routes, resulting in the production of different types of MVs. Methods such as high-speed atomic force microscopy (AFM) phase imaging allow the physical properties of MVs to be analyzed at a nanometer resolution, revealing their heterogeneity. In this special minireview, we introduce the role of MVs in bacterial communication and highlight recent findings on MV formation and their physical heterogeneity by referring to our research. We hope that this minireview will provide basic information for understanding the functionality of MVs in ecological systems.

## Bacterial communication

Bacteria communicate with each other through signaling molecules and coordinate group behavior by altering gene expression. One of the most studied signaling molecules in Gram-negative bacteria is *N*-acyl homoserine lactone (AHL), which is involved in a cell-density-dependent signaling system known as quorum sensing. The chemical structure of AHLs consists of a lactone ring and acyl side chain with lengths ranging between 4 and 20 carbons, which may be modified (Arashida *et al.*, 2018). These signals are specifically recognized by their cognate LuxR-type receptors and often regulate genes involved in virulence factor, biosurfactant, and biofilm formation (Schuster *et al.*, 2013). One of the key steps in bacterial communication is the spread of signaling molecules, which eventually reach other cells. While short-chain AHLs have been shown to diffuse from cells, long-chain AHLs are hydrophobic and mostly partition with the cell envelope (Blosser-Middleton and Gray, 2001; Marketon *et al.*, 2002; Schaefer *et al.*, 2002; Wagner-Döbler *et al.*, 2005); therefore, further studies are warranted to elucidate the mechanisms by which these hydrophobic molecules function as a signal.

## Signal delivery through membrane vesicles (MVs)

Recent studies demonstrated that membrane vesicles (MVs) play an important role in the release of hydrophobic molecules, including signaling molecules, from cells and their dispersal in aquatic environments. MVs typically range between 20 and 400‍ ‍nm in diameter and mainly consist of cellular membranes. They contain various types of cargo, such as genetic material (DNA or RNA) and toxins, which are involved in diverse bacterial and bacteria-host interactions (Kaparakis-Liaskos and Ferrero, 2015; Toyofuku *et al.*, 2019). MVs have also been used as platforms for vaccine development and as drug delivery systems (Toyofuku *et al.*, 2015; Zhu *et al.*, 2021). Initial research on MVs in Gram-negative bacteria showed that they formed through the blebbing of the outer membrane (Chatterjee and Das, 1967; Devoe and Gilchrist, 1973). These outer membrane vesicles (OMVs) are mainly produced by the overproduction of the membrane, the loss of the linkage between the inner and outer membranes, and increased turgor pressure in the periplasm (Toyofuku *et al.*, 2019). Furthermore, the intercalation of molecules into the outer membrane results in blebbing. Blebbing has been observed in *Pseudomonas aeruginosa*, which produces a hydrophobic quinolone signal known as the* Pseudomonas* quinolone signal (PQS), in addition to two types of AHLs, C4-HSL and 3-oxo-C12-HSL (Pesci *et al.*, 1999). PQS is a multifunctional molecule that chelates iron and inhibits respiratory activity in addition to its function as a signaling molecule (Bredenbruch *et al.*, 2006; Toyofuku *et al.*, 2008). Similarly, PQS has been shown to induce the formation of OMVs in *P. aeruginosa* independent of signal receptors without regulating gene expression (Mashburn and Whiteley, 2005). Further studies demonstrated that the insertion of PQS led to the expansion of the outer leaflet of the outer membrane, resulting in the formation of OMVs through blebbing (Schertzer and Whiteley, 2012). PQS molecules are then dispersed and delivered by OMVs, which activate the QS response in *P. aeruginosa*. In addition to PQS, other signaling molecules, such as CAI-1 in *Vibrio harveyi* (Brameyer *et al.*, 2018) and long-chain AHLs in *Paracoccus* species (Toyofuku *et al.*, 2017b; Morinaga *et al.*, 2018; Morinaga *et al.*, 2020), both of which are hydrophobic, are delivered through MVs. Using a bioreporter that responds to AHLs, MVs derived from the coral pathogen *V. shilonii* were found to contain AHLs, the structure of which remains unclear (Li *et al.*, 2016). Another group of well-studied hydrophobic signaling molecules are diffusible signal factor (DSF) family molecules, at least two of which are carried by MVs in the plant pathogen *Xanthomonas fastidiosa* (Feitosa-Junior *et al.*, 2019). In *X. fastidiosa*, MV formation was shown to be suppressed by a DSF family molecule (Ionescu *et al.*, 2014). Since MVs inhibited the attachment of *X. fastidiosa* to xylem vessels, the DSF-induced suppression of MV formation stimulated biofilm formation on plant surfaces (Ionescu *et al.*, 2014). In the case of *Stenotrophomonas maltophilia*, DSF induced the formation of MVs involved in the secretion of the virulence factor protein Ax21, which is a membrane protein (Devos *et al.*, 2015). The production of DSF family molecules has been reported in several bacteria; however, limited information is currently available on the mechanisms by which they diffuse in aquatic environments. Therefore, further studies are warranted to establish whether these molecules are carried by MVs.

## MV-mediated bacterial communication in *Paracoccus* species

*Paracoccus denitrificans* produces a hydrophobic long-chain AHL, *N*-hexadecanoyl homoserine lactone (C16-HSL), the synthetic enzyme of which is encoded by *pdnI* (Toyofuku *et al.*, 2017b). This non-motile bacterium forms a thin-layered biofilm that is largely dependent on the surface adhesion protein, biofilm-associated protein A (BapA) (Yoshida *et al.*, 2017). During a planktonic culture, *pdnI* mutant aggregates are inhibited by the addition of C16-HSL. MVs carrying C16-HSL, but not those derived from the *pdnI* mutant, inhibit cell aggregation, indicating that MVs deliver C16-HSL. Quantitative data showed that C16-HSL was enriched in MVs at a high concentration, which may trigger the QS response of a single cell (Toyofuku *et al.*, 2017b). In other words, a “single shot” of MV may trigger the QS response in a cell. MV-assisted QS would lead to a mode of signaling in which the QS response is only triggered in cells that receive MVs, the mechanism of which is referred to as binary signaling (Toyofuku *et al.*, 2017b), whereas the classical QS model assumes the free diffusion of signals that synchronize gene expression in the population. Other *Paracoccus* species have also been reported to release long-chain AHLs via MVs (Morinaga *et al.*, 2020). One exception is *Paracoccus aminophilus*, which produces C16-HSL and C18-HSL, which are not mainly associated with MVs. The detection of these non-MV-associated AHLs in *P. aminophilus* and other strains suggests the presence of unidentified long-chain AHL carriers.

## Signal interception by MVs

In addition to releasing hydrophobic molecules from cells, MVs adsorb signal molecules from the environment (Morinaga *et al.*, 2018). This may allow bacteria to intercept QS signals from other bacterial species. *P. denitrificans* mainly uses C16-HSL as its own signal, but may also respond to C12-, C14-, and C18-HSLs. When each signal is incubated together with MVs derived from a *P. denitrificans pdnI* mutant, the signal is loaded onto MVs at a concentration that triggers the QS response in *P. denitrificans*. The cell specificity of MVs is an important factor for intercepting signals; however, it has not yet been examined in detail. When the MVs of *P. denitrificans* were added to a mixture of *P. denitrificans* and *P. aeruginosa*, *P. denitrificans* received MVs, whereas *P. aeruginosa* did not (Toyofuku *et al.*, 2017b). Further studies showed that the cell-specific delivery of MVs was involved in gene transfer and iron acquisition (Lin *et al.*, 2017; Tashiro *et al.*, 2017; Li *et al.*, 2022). The mechanisms by which the MVs of *P. denitrificans* are primarily received by their own species remain unclear. However, *P. denitrificans* has been shown to respond to MVs produced by other *Paracoccus* species (Morinaga *et al.*, 2020), suggesting that MVs play important roles in both intra- and inter-species interactions.

## Explosive cell lysis stimulates signal release through MV formation

**Fig. 1.**
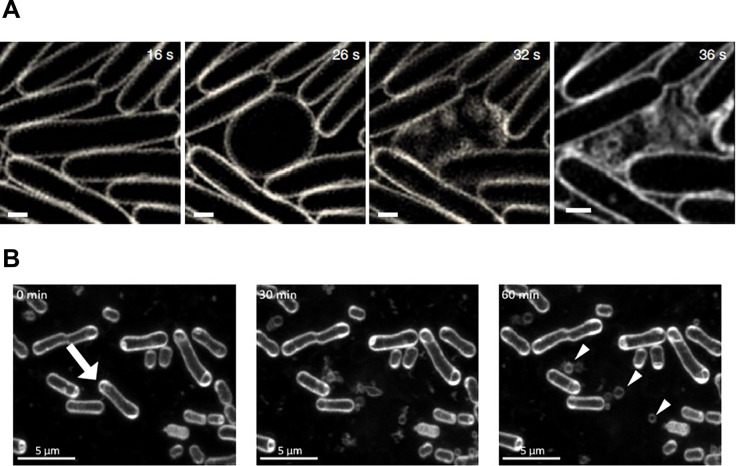
Explosive cell lysis in *Pseudomonas aeruginosa* (A) and *Paracoccus denitrificans* (B). Scale bars indicate 0.5 and 5‍ ‍μm, respectively. Reprinted with permission from Turnbull *et al.* (2016) and Yasuda *et al.* (2022).

MV formation in *P. denitrificans* is poorly understood; however, a recent study showed that at least genes encoded in the prophage region induced MV formation through explosive cell lysis, in which MVs were also found to be highly enriched in phage DNA (Yasuda *et al.*, 2022). Explosive cell lysis was initially demonstrated in *P. aeruginosa*, in which peptidoglycan was degraded by endolysin, resulting in cells shattering their membrane, which then rounded up to form MVs (Turnbull *et al.*, 2016). Endolysin is widely conserved among bacteria, is typically encoded in the prophage region of dsDNA phages, and plays crucial roles in phage release from the host cell. It is regulated by RecA, which is a key regulator of the SOS response and is activated by DNA breakage. Endolysin works in pairs with a holin to create a hole in the membrane, thereby allowing endolysin to access peptidoglycan (Cahill and Young, 2019). As cells lyse, their cytoplasmic contents are released, including DNA, which function as the extracellular matrix in biofilm formation (Turnbull *et al.*, 2016). *P. aeruginosa* changes from rod-shaped to round cells prior to lysis (Turnbull *et al.*, 2016), whereas *P. denitrificans* mainly lyses as rods (Yasuda *et al.*, 2022), suggesting that both are triggered by peptidoglycan degradation; however, this process differs among species (Fig. 1). The effects of the different processes of explosive cell lysis on the composition of MVs has yet to be clarified. Explosive cell lysis in *P. denitrificans* was previously shown to stimulate the release of C16-HSL due to an increase in the formation of MVs (Yasuda *et al.*, 2022). This finding indicates that a prophage gene interferes with bacterial communication through MV formation, providing insights into the interplay between phages and bacteria. Previous studies showed that MVs play different roles in the phage-bacteria interplay and that virulent phages also induce the formation of MVs (Tzipilevich *et al.*, 2017; Mandal *et al.*, 2021). However, it currently remains unclear whether prophage-induced MV formation benefits the host or phage in *P. denitrificans*.

## Cell wall damage triggers MV formation in Gram-positive bacteria

The involvement of endolysins in the formation of MVs has been reported in phylogenetically distinct species, including Gram-positive bacteria. In *Bacillus subtilis*, endolysin was found to trigger cytoplasmic membrane vesicle (CMV) formation through bubbling cell death, during which it created a hole in the cell wall, causing the cellular membrane to protrude and be released as CMVs (Toyofuku *et al.*, 2017a). The holin-endolysin system also triggers CMV formation in *Lactococcus species, Staphylococcus aureus* (Andreoni *et al.*, 2019; da Silva Barreira *et al.*, 2022; Liu *et al.*, 2022), and other Gram-positive bacteria that possess this system. Similarly, autolysins have been shown to induce CMV formation in *B. subtilis* through bubbling cell death, which supports cell wall damage or modifications being one of the key steps in CMV formation in Gram-positive bacteria (Abe *et al.*, 2021). Consistent with these findings, antibiotics that inhibit cell wall synthesis were found to induce the formation of MVs through bubbling cell death and other mechanisms that involve blebbing of the membrane (Wang *et al.*, 2018; Andreoni *et al.*, 2019; Nagakubo *et al.*, 2021). Therefore, the treatment of bacteria with specific antibiotics that inhibit cell wall synthesis or trigger the SOS response and induce the expression of prophage genes may induce MV formation and neutralize membrane-targeting antibiotics (Andreoni *et al.*, 2019).

## Heterogeneity of MVs

**Fig. 2.**
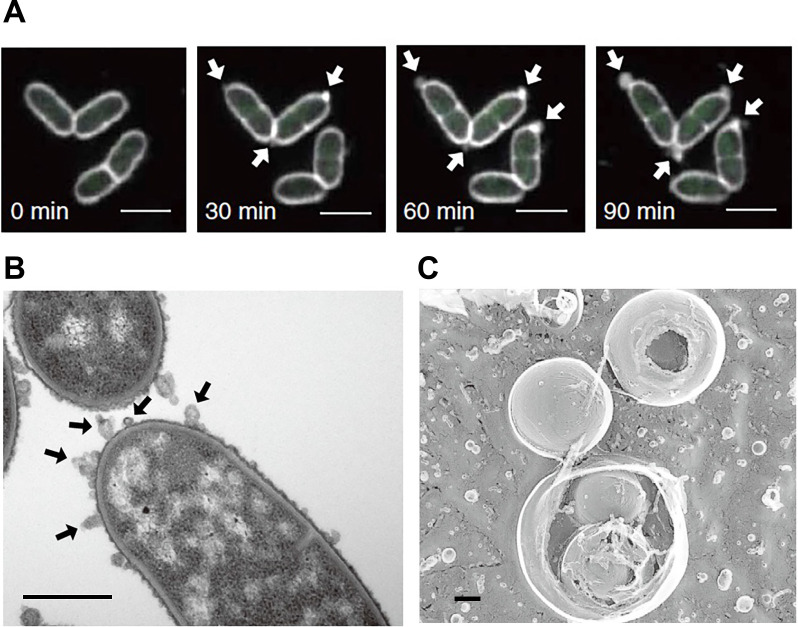
Membrane vesicle (MV) formation in penicillin G-treated *Corynebacterium glutamicum*. MVs form at the cell poles of cells treated with penicillin G (A and B). Multivesicular structures in MVs derived from *C. glutamicum* treated with penicillin G (C). Scale bars, 2, 1, and 0.2‍ ‍μm, respectively. Reprinted with permission from Nagakubo *et al.* (2021).

Previous studies indicated that different routes for MV formation gave rise to different types of MVs (Toyofuku *et al.*, 2019). Blebbing in Gram-negative bacteria mainly leads to the generation of OMVs, whereas explosive cell lysis gives rise to outer-inner membrane vesicles (OIMVs) and explosive outer-membrane vesicles (EOMVs) (Toyofuku *et al.*, 2019). Due to the presence of the inner membrane, OMVs are devoid of cytoplasmic material, whereas OIMVs and EOMVs contain cytoplasmic contents, such as DNA. CMVs are formed by Gram-positive bacteria, whereas mycolic acid-containing bacteria, a group of Gram-positive bacteria that possess an extra layer of the mycomembrane outside the cell wall, generate different types of MVs (Nagakubo *et al.*, 2021). Due to their complex cell envelope structure, mycolic acid-containing bacteria form MVs through blebbing and bubbling cell death, giving rise to both OMVs and CMVs, which are also referred as mycomembrane vesicles (mMVs) and inner membrane vesicles (IMVs), respectively. It currently remains unclear whether OIMVs are formed. In *Corynebacterium glutamicum*, localized MV formation at the cell poles was observed in cells treated with penicillin G (Nagakubo *et al.*, 2021) (Fig. 2A and B). This may be attributed to active cell wall synthesis at the cell poles (Sher *et al.*, 2021). OMVs generated from the cell poles are rich in cardiolipin, a phospholipid that segregates into regions with a high membrane curvature, indicating that the cellular site of MV formation affects their composition (Nagakubo *et al.*, 2021). OMVs formed by penicillin G in *C. glutamicum* showed a unique multivesicular structure that was not observed in other types of MVs of this strain (Fig. 2C). Based on the heterogeneity of MVs and the potential of a single MV particle to influence cell physiology, it is important to examine MVs in more detail at the single particle level.

## Analysis of physical properties of single MV particles using high-speed atomic force microscopy (AFM)

**Fig. 3.**
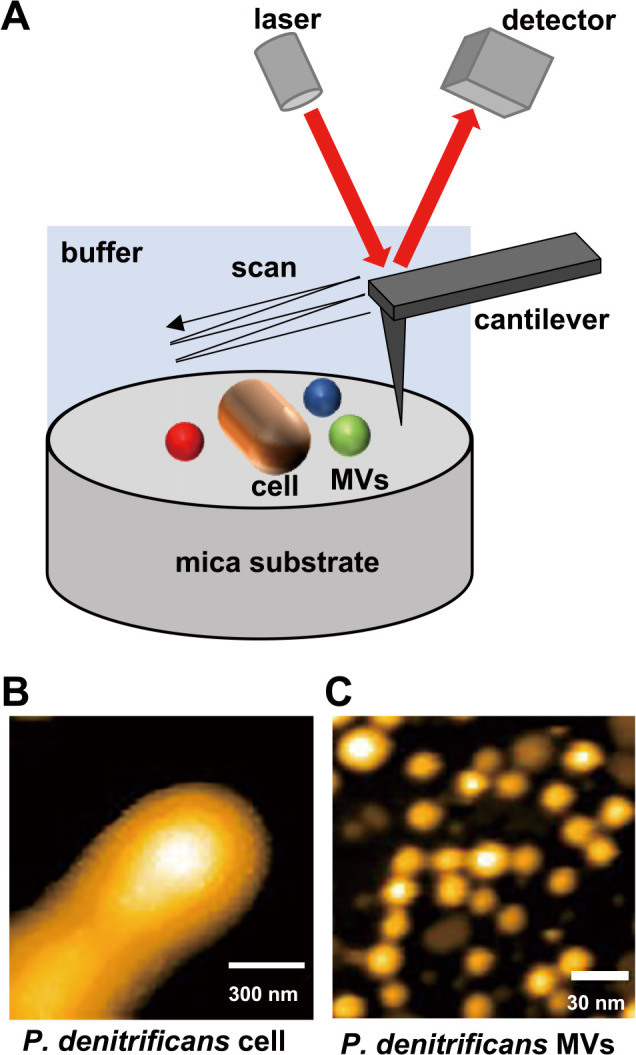
(A) Schematic diagram of high-speed atomic force microscopy (AFM) observations. AFM topographic images of the living cell surface (B) and MVs of *Paracoccus denitrificans* (C). Reprinted with permission from Seibutsu-kogaku Kaishi 98: 357–360, 2020.

High-speed AFM is a promising method for analyzing MVs. AFM generates three-dimensional images based on the force detected between a sample surface and a small sharp tip (probe) in a liquid, such as buffer solution or medium (Fig. 3). Therefore, AFM visualizes the structures of electron-permeable biological molecules, including proteins, nucleic acids, lipids, and complexes of these molecules, under physiological conditions with nanometer spatial resolution. AFM has been successfully applied not only to the imaging of purified specimens, but also to the imaging and ana­lysis of live cells (Xiao and Dufrêne, 2016; Dufrêne *et al.*, 2017). High-speed AFM has been developed to image the structure and dynamics of fragile biological specimens (Uchihashi *et al.*, 2012; Ando, 2017, 2018). The maximum force applied to samples during high-speed AFM observations is 100 pN, which is sufficiently low for non-destructive observations of soft biological samples; therefore, it is ideal for investigating the dynamics of protein molecules, such as myosin V, F1-ATPase, and GroEL–GroES (Ando, 2018). Moreover, high-speed AFM is used to image structural changes on the diverse surfaces of living cells, including HeLa cells (Shibata *et al.*, 2015) and bacterial cells, including *Magnetospirillum magneticum* AMB-1 (Yamashita *et al.*, 2012), *Rhodobacter sphaeroides*, and *Escherichia coli* (Oestreicher *et al.*, 2015). It has also been used to image the gliding machinery of *Mycoplasma mobile* cells (Kobayashi *et al.*, 2021).

AFM has multiple observation and measurement modes, such as force-distance curve-based AFM, chemical force microscopy, mole­cular recognition AFM, and multi-frequency AFM (Dufrêne *et al.*, 2017). These are useful for imaging or analyzing the distribution of the physical properties and chemical compositions of sample surfaces. The phase mode is one of the AFM imaging modes that allows for the mapping of surface physical properties, such as adhesion, elasticity, and/or friction, at a nanometer spatial resolution (Garciá, 2010). This AFM mode is frequently used in the field of surface and material sciences (Magonov and Reneker, 1997; Connell and Smith, 2006). However, its application to biological specimens has been limited.

**Fig. 4.**
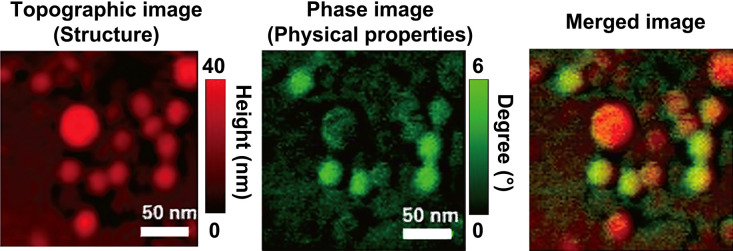
High-speed AFM phase images of *Paracoccus denitrificans* MVs. Topographic and phase images show the structures and physical properties of individual MVs, respectively. In the merged image, MVs with low and high adherence/elasticity are shown as reddish and greenish particles, respectively. Reprinted with permission from Seibutsu-kogaku Kaishi 98: 357–360, 2020.

The high-speed AFM phase mode was previously used to quantitatively analyze the surface physical properties of single MV particles in solution (Kikuchi *et al.*, 2020). MV particles isolated from *P. denitrificans* cultures were immobilized on a 3-aminopropyltriethoxysilane-treated mica substrate and observed using AFM in phosphate-buffered saline (PBS). Fig. 4 shows an example of the topography and phase images of *P. denitrificans* MVs. In the phase image, the color shows differences in the phase-shift values that reflect the intensities of the force between the sample surface and AFM tip. The colors of individual MV particles are different. The merged topography and phase image shows that reddish and greenish MV particles exhibited low and high adherence/elasticity, respectively. These findings indicate that *P. denitrificans* released MV particles with heterogeneous physical properties even under normal culture conditions. To compare MVs from different bacterial species, a method for quantitatively analyzing the physical properties of individual MVs was developed in the same study. Polystyrene microbeads and mica surface were used as internal standards to normalize phase-shift values, thereby allowing comparisons of different samples. MVs derived from four species, including three Gram-negative bacteria (*E. coli*, *P. aeruginosa*, and *P. denitrificans*) and one Gram-positive species (*B. subtilis*), were examined, and the findings obtained showed that all four species released MVs with heterogeneous physical properties. Moreover, the distribution of the physical properties of MVs showed species-specific patterns. MVs produced by *E. coli* and *P. denitrificans* exhibited higher absorptivity than those produced by the other two species. Further studies are needed to establish how these physical properties reflect MV contents and functionality, including the cell-specific delivery of MVs.

## Conclusions

The diversity of MVs is becoming evident as we continue to elucidate the mechanisms underlying their formation. MVs may be classified into several groups according to their origin and route of formation. However, since limited information is currently available on the characteristics of distinct types of MVs, the functions of individual particles appear to have been averaged out. For example, in bacterial communication, a single MV may alter the gene expression of a cell, providing some indication of the latent potential of single MVs. While the accumulation of information on MV contents and functions is continuing, the interactions between MVs and cells, and, thus, the functionality of MVs have yet to be elucidated in detail. Physical properties, which are heterogeneous among vesicles (Kikuchi *et al.*, 2020), may be an important factor influencing their functionality (Tashiro *et al.*, 2017). Due to this heterogeneity, future research that tracks the fate of an MV will provide insights into the functionality of MVs.

## Citation

Toyofuku, M., Kikuchi, Y., and Taoka, A. (2022) A Single Shot of Vesicles. *Microbes Environ ***37**: ME22083.

https://doi.org/10.1264/jsme2.ME22083
